# Microbiome Analysis Reveals the Attenuation Effect of *Lactobacillus* From Yaks on Diarrhea *via* Modulation of Gut Microbiota

**DOI:** 10.3389/fcimb.2020.610781

**Published:** 2021-02-16

**Authors:** Hailong Dong, Bingxian Liu, Aoyun Li, Mudassar Iqbal, Khalid Mehmood, Tariq Jamil, Yung-Fu Chang, Hui Zhang, Qingxia Wu

**Affiliations:** ^1^ Animal Science College, Tibet Agriculture & Animal Husbandry University, Linzhi, China; ^2^ College of Veterinary Medicine, South China Agricultural University, Guangzhou, China; ^3^ College of Veterinary Medicine, Huazhong Agricultural University, Wuhan, China; ^4^ Faculty of Veterinary and Animal Sciences, The Islamia University of Bahawalpur, Bahawalpur, Pakistan; ^5^ Institute of Bacterial Infections and Zoonoses, Friedrich-Loeffler-Institut, Jena, Germany; ^6^ Department of Population Medicine and Diagnostic Sciences, College of Veterinary Medicine, Cornell University, Ithaca, NY, United States

**Keywords:** Tibet Plateau, yak, gut microbiota, *Lactobacillus*, *Escherichia coli*

## Abstract

Domestic yaks (*Bos grunniens*) are indigenous to the Tibetan Plateau and display a high diarrhea rate due to poor habitat and husbandry conditions. *Lactobacillus* has been shown to exert beneficial effects as antimicrobial, growth promotion, and gut microbiota in humans and/or murine models, but the relevant data regarding *Lactobacillus* isolated from yaks was unavailable. Therefore, this study aimed to investigate the effects of *Lactobacillus* from yaks on the intestinal microbial community in a mouse model and determine whether *Lactobacillus* supplementation contributed in alleviating diarrhea by modulating gut microbiota. A total of 12 ileac samples from four groups were collected for 16S rRNA gene amplicon sequencing of V3-V4 region. Results revealed that although *Lactobacillus* supplementation did not change the diversity of gut microbiota in mice, the proportion of some intestinal microbiota significantly changed. Specifically, the proportion of *Lactobacillus* and *Sphingomonas* in the *Lactobacillus* treated-group (L-group) were increased as compared to control group (C-group), whereas *Pantoea*, *Cutibacterium*, *Glutamicibacter*, *Turicibacter*, *Globicatella*, *Microbacterium*, *Facklamia*, *unidentified_Corynebacteriaceae*, *Brachybacterium*, and *Staphylococcus* were significantly decreased in the L-group. In contrast, *Escherichia coli* (*E. coli*) infection significantly decreased the proportion of beneficial bacteria such as *Globicatella*, *Acinetobacter*, *Aerococcus*, and *Comamonas*, while loads of pathogenic bacteria significantly increased including *Roseburia* and *Megasphaera*. Interestingly, *Lactobacillus* administration could ameliorate the microbial community structure of *E. coli*-induced diarrheal mice by reducing the relative abundance of pathogenic bacteria such as *Paenibacillus*, *Aerococcus*, *Comamonas*, *Acinetobacter*, *Corynebacterium*, *Facklamia*, and *Globicatella*. Results in this study revealed that *Lactobacillus* supplementation not only improved the gut microbiota but also alleviated diarrhea in mice, which may be mediated by modulating the composition and function of gut microbiota. Moreover, this study is expected to provide a new theoretical basis for the establishment of a preventive and treatment system for diarrhea in yaks.

## Introduction

Animal gut microbiota is one of the largest and most complicated existing micro-ecosystems that provides an important barrier to bacterial infections (Lynch and Pedersen, 2016; Li et al., 2020; Liu et al., 2020b). Additionally, it helps in providing mucosal immunity, material metabolism, and nutrient absorption and regulation (Wu and Wu, 2012; Yue et al., 2020). Generally, ongoing competition and interaction of microorganisms may gradually change in microbial community structure from simple to a complicated and eventually a dynamic and balanced ecosystem (Jami et al., 2013; Zhao et al., 2015). This community’s consistency is a precondition for maintaining normal physiological functions (Li et al., 2018a; Ritz et al., 2020). Previous research has shown that constipation, colitis, diabetes, and obesity may be related to alternation in intestinal flora (Cani et al., 2008; de La Serre et al., 2010; Kootte et al., 2012). Recent studies on gut microbiota have provided evidence that dysbacteriosis may be one of the reasons of diarrhea (Han et al., 2017; Yue et al., 2019).

Domestic yak (*Bos grunniens*) is an indigenous breed of the high-elevation hypoxic environment in the Tibetan plateau of China (3000 m above sea level or higher), spanning Mongolia and Siberia (Li K. et al., 2014; Li et al., 2018a). This biome is characterized by plants and animals adapted to cold and dry adverse environments (Qiu et al., 2012). Statistics indicate that approximately 90% of the word’s yaks reside in western China, where this animal is an important source of milk, meat, and draft power (Li et al., 2017; Li et al., 2019a). Any infectious disease in these yaks may result in substantial economic losses to the herdsmen relying on them. However, due to a lack of corresponding supervision and weak awareness of environmental issues, yaks (particularly juveniles) in Tibet typically display higher incidence of bacterial diarrhea (Gao et al., 2013; Han et al., 2017).

Diarrhea poses a significant threat to animal productivity in many countries (Pepin et al., 2004; Heuer et al., 2007). Statistical analyses indicate that yaks, especially juveniles, display a higher prevalence of diarrhea due to a lack of corresponding protocols and practices designed to sustain animal health (Pepin et al., 2004; Heuer et al., 2007). The aggressive use of antimicrobials as therapeutic agents has resulted in increased drug resistance and intestinal microbial imbalance (van den Bogaard and Stobberingh, 2000; Zhang et al., 2017). The importance of probiotics including *Lactobacillus johnsonii*, *Bacillus subtilis*, and *Pediococcus acidilactici* have been proverbially acknowledged because of their effects as potential antibacterial, immunity, and production enhancing performances (Li et al., 2018; Wang et al., 2018a; Khan, 2019; Li et al., 2019b). Moreover, *Lactobacillus* can alleviate bacterial diarrhea by competing with pathogenic bacteria for adhesion sites and nutrients, producing antimicrobial peptides, and improving gut microbiota (Sadowska et al., 2010; Huang et al., 2020; Wang et al., 2020). The probiotic effect of *Lactobacillus* has been understood and applied in chicken and other domestic animals. In yaks, probiotic applications are not well studied, so this species is a relatively new subject for probiotic research (Dudik et al., 2020: Sobanbua et al., 2020). Given the importance of yak husbandry in the Tibetan plateau, such research might be a worthy endeavor. Taking advantage of this gap, we investigated the effects of *Lactobacillus* (isolated from yaks) systematically on mice’s gut microbiota. Additionally, we also established diarrheal model using *E. coli* to identify *Lactobacillus* administrative effect on the intestinal microbiota in diarrheal mice.

## Materials and Methods

### Animal Experiments and Sample Collection

Twenty-five-day-old healthy Kunming mice (n=40, initial weight 30 ± 3 g) were obtained from an experimental animal center, South China Agricultural University (Guangzhou, China). The study was permitted by the ethics committee of Tibet Agriculture & Animal Husbandry University. The mice used in this study were self-propagated and showed a higher degree of genetic uniformity. The selected mice were randomly divided into four groups, each comprising 10 mice (n=10) viz. control group (C-group), *Lactobacillus*-treated group (L-group), *E. coli*-induced group (E-group), and prevention group (EL-group). The mice were raised in plastic cages for 14 days under a recommended standard illumination time (12 h/12 h light/dark cycle), breeding temperature (33°C~35°C), and humidity (53%–57%). Furthermore, sufficient water and feed were provided *ad libitum* for all groups throughout the entire experiment. The E group was provided with same diet as control group but with the addition of *E. coli* at 1 × 10^9^ CFU/day on day 14 post-hatch to induce diarrhea. The L and EL group were treated with *Lactobacillus* at 1 × 10^9^ CFU/day from day 1 to day 14, but mice in the EL group were compulsively supplemented with *E. coli* at 1 × 10^9^ CFU/day on day 14. Mice in the C group were provided with the same volumes of saline as the L group to minimize stress response. Moreover, the overall performance of mice was recorded three times a day and death, diarrhea, dullness, and tiredness were considered abnormal. Three mice from each group were euthanized by injecting pentobarbital (25 mg/kg). Subsequently, the intestines were removed from the abdominal cavity, and the mesentery was stripped using a sterilized surgical knife. The intestines including duodenal, ileum, jejunal, and cecum were knotted using cotton ropes to minimize the potential cross-contamination among different intestine samples. The contents in the ileum were immediately collected, snap-frozen using liquid nitrogen, and finally stored at -80°C until further study.

### gDNA Extraction

Bacterial DNA from all samples was extracted by using QIAamp DNA Mini Kit (Qiagen, Hilden, Germany) as per manufacturer’s guidelines. The quality of DNA was evaluated by 0.8% (w/v) agarose gel electrophoresis. A Nanodrop™ Spectrophotometer (Thermo Scientific, Massachusetts, USA) was used to quantify the DNA.

### 16S rRNA Gene Amplification and Sequencing

Universal V3/V4 16S rRNA gene primers (338F: ACTCCTACGGGAGGCAGCA and 806R: GGACTACHVGGGTWTCTAAT) were used along with the barcode sequences for amplification of the conserved regions of the bacteria followed by a 2% agarose gel electrophoresis procedure to evaluate the quality of the polymerase chain reaction (PCR) products. The PCR product was then purified and recycled by using AxyPrep DNA Gel Extraction Kit (Axygen, Corning, New York, USA). The fluorescent quantitation of PCR-recycled products was conducted on FLx800™ Multi-Detection Microplate Reader (BioTek Instruments, Inc., Winooski, Vermont, USA) by using Quant-iT™ PicoGreen™ dsDNA Assay Kit (Invitrogen, Carlsbad, California, USA). The sequencing library was prepared by using TruSeq Nano DNA Low Throughput Library Prep Kit (Illumina, Inc., San Diego, California, USA) following the manufacturer’s protocol. The amplified products were repaired by End Repair Mix. Simultaneously, a magnetic bead screening procedure was used for removing the self-connecting fragments of the linker, and the sequencing library was purified. The PCR amplification was performed, and the library enrichment was performed by AMPure XP Beads (Beckman Coulter Inc., Brea, California, USA). The final fragment-selection and purification of the library were conducted on 2% agarose gel electrophoresis.

The libraries’ quality was accessed by using Agilent High Sensitivity DNA Kit on Agilent Bioanalyzer (Agilent Technologies, Inc., Santa Clara, California, USA) prior to the sequencing process. Moreover, the libraries having only one single peak without a linker was selected. The libraries were quantified by using Quant-iT PicoGreen dsDNA Assay Kit (Invitrogen, Carlsbad, California, USA) on the QuantiFluor^®^ RNA System (Promega Corporation, Madison, Wisconsin, USA), with a concentration >2 nM. The qualified libraries were gradient diluted and mixed in a proportion to the required amount of sequencing. Finally, the MiSeq Reagent Kit V3 (600 cycles) was used to perform the 2×300 bp paired-end sequencing on the MiSeq sequencing system (Illumina, Inc., San Diego, California, USA).

### Bioinformatics and Statistical Analysis

QIIME software (Qiime1.9.1) was used to screen and analyze the 16S rRNA preliminary data quality. The interrogative and short sequences (<200 bp) were removed by using QIIME software. The obtained sequences were clustered and operational taxonomic unit (OTU) were partitioned at ≥97% sequence similarity by program VSEARCH (1.9.6). The Ribosomal Database Program (RDP) classifier was used to classify the representative sequences of each OTU at confidence threshold of 0.8. The MUSCLE software was used for phylogenetic analysis and multiple sequence alignments of each OTU. The multiple alpha diversity indices including Shannon, Simpson, Chao1, and Good’s coverage were calculated to evaluate the alpha diversity. Moreover, the sparse curves were used for assessing the sequencing depth of each sample prior to the evaluation of alpha and beta diversity. The beta diversity was also calculated to assess the similarity of community structure in the samples. GraphPad Prism (version 6.0c) and R (v3.0.3) software were used to perform the statistical analysis. In addition, the criterion of significance was conducted at p-values <0.05. The values were expressed as means ± standard deviation (SD).

## Results

### Clinical Symptoms

Clinical observation results showed that the mice in the C and L group had a normal feed intake and displayed active behavior. However, mice in the E group showed dullness, messy hair, watery feces, and pasting. Conversely, mice in the EL group and the control group were in good mental state and without diarrhea symptoms.

### DNA Sequences Analyses

**Figure 1:**
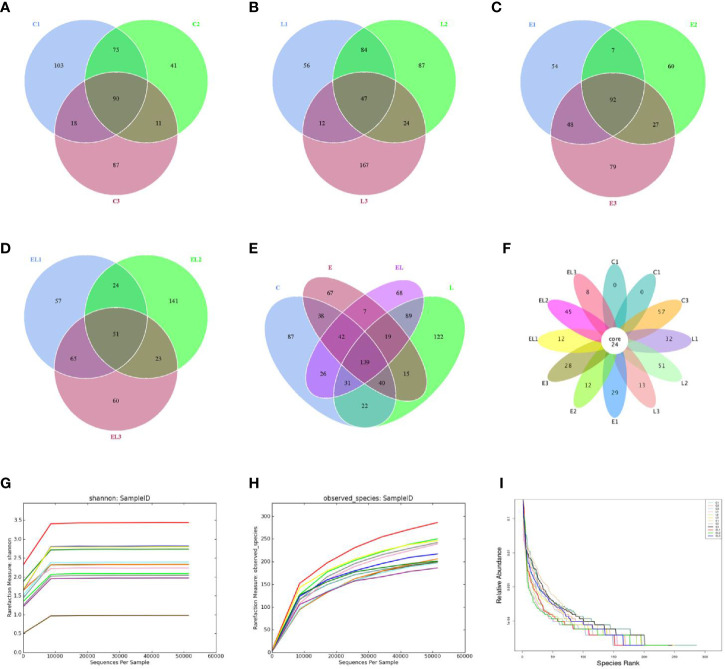
Venn diagrams and sample feasibility analysis. Venn diagrams for bacterial OTUs compositions in C **(A)**, L **(B)**, E **(C)** and EL **(D)** groups. **(E)** Venn diagram for unique and shared bacterial OTUs in four groups. **(F)** Venn diagrams for core OTUs compositions. The rarefaction **(G)** and species accumulation curve **(H)** and rank abundance curve **(I)** were used to assess the adequacy, evenness and richness of sequencing of each sample. Each curve with a different color shown in the figures indicates a sample.

In the microbiome analysis, a total of 274,247, 265,341, 263,510, and 253,825 original sequences were acquired from C, L, E, and EL-group, respectively (**Table 1**). After eliminating the unqualified data, a total number of 961,904 high-quality reads were achieved from all the samples, with an average of 80,158 (ranging from 75,598 to 90,528) reads per sample. Following taxonomic assignment, a total of 2,668 OTUs (C=709, L=691, E=633, EL=635) were identified on the basis of 97% nucleotide-sequence similarity and 139 OTUs have shared all the samples, accounting for approximately 5.21% of the total OTUs (**Figures 1A–E** **)**. Moreover, the number of unique OTUs in the C, L, E, and EL group was 87, 122, 67, and 68, respectively and 16 core OTUs were recognized in all the samples (**Figure 1F**). Both rarefaction and species accumulation curves for all samples tend to be stable. The number of qualified sequences reached over 10,000 and 50,000, respectively, suggesting that sequencing’s depth and quantity met the demands for sequencing and analysis (**Figures 1G, H** **)**. Furthermore, the rank abundance curve is wide and falling relaxedly, showing excellent abundance and evenness (**Figure 1I**).

**Table 1:** The sequence information of each sample.

Sample	Raw_reads	Combined_reads	Qualified_Reads	Effective (%)
C1 C2 C3 L1 L2 L3 E1 E2 E3 EL1 EL2 EL3	87,037 89,587 97,623 85,953 94,888 84,500 83,003 84,412 96,095 89,464 80,217 84,144	79,283 85,791 81,093 83,188 92,730 80,291 78,273 81,212 90,371 85,212 77,592 81,085	76,082 82,857 76,128 80,494 90,528 77,475 76,118 78,706 87,082 82,185 75,598 78,651	87.41% 92.49% 77.98% 93.65% 95.42% 91.69% 91.71% 93.24% 90.62% 91.86% 94.24% 93.47%

### Microbial Diversity Index in Different Groups

**Figure 2:**
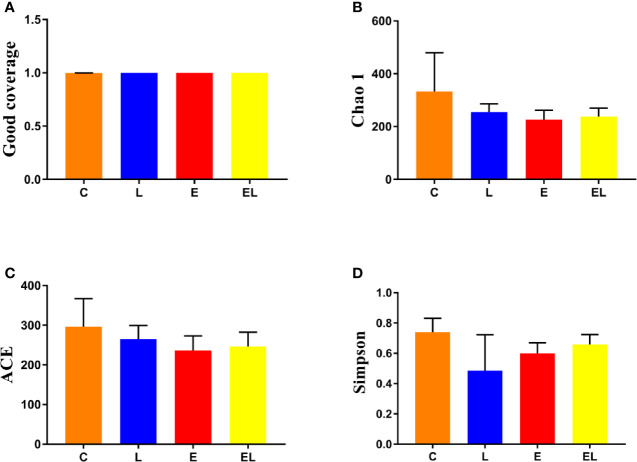
Comparison of alpha diversity of mice gut microbiota in different groups. Four indices such as Good's coverage **(A)**, Chao1 **(B)**, ACE **(C)**, and Simpson **(D)** were used to assess the alpha diversity of gut microbiota. The data used in this study were expressed as the mean ± SD.

**Figure 3:**
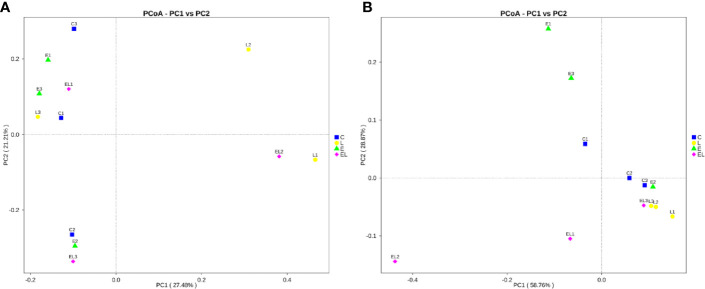
Principal coordinate (PCoA) analysis of gut microbiota in different groups. **(A, B)** represent PCoA map based on unweighted and weighted uniFrac distance, respectively. Each colored point indicates one sample and the difference in the different groups can be evaluate by the distance between the points.

To assess the differences in intestinal microbial community diversity among the four groups, the qualified sequences obtained in the sequencing were aligned to estimate alpha and beta indices. The alpha diversity of gut microbiota can be reflected by community abundance (Chao1 and ACE), diversity index (Simpson), and sequencing depth (Good’s coverage). Good’s coverage estimates in all the samples were approximately 100%, indicating excellent coverage (**Figure 2A**). The control mice showed the highest Chao1 and ACE indexes as compared to other groups, whereas the Chao1 and ACE indexes in mice infected with *E. coli* were the lowest (**Figures 2B, C** **)**. The average of Chao1 and ACE indices in the L-group ranged from 218.71 to 277.02 and 226.022 to 289.15, respectively, while Simpson index ranged from 0.22 to 0.62. The analysis of alpha diversity indicated no statistically significant differences in the Chao1, ACE, and Simpson between the C and L groups, which indicated that *Lactobacillus* administration had no effect on the diversity and richness of the gut microbiota of mice (**Figure 2D**). However, intergroup analysis of alpha diversity intuitively indicated that gut microbiota’s richness and diversity in EL-group mice were higher than those in E-group, indicating that supplementing with *Lactobacillus* alleviated the gut microbiota imbalance of mice induced by *E. coli*. The beta diversity analysis showed that samples in the C, L, E, and EL groups were clustered closely, indicating that gut microbiota in the four groups was not different (**Figure 3**).

### Alterations in the Composition of Gut Microbiota in Different Groups

**Figure 4:**
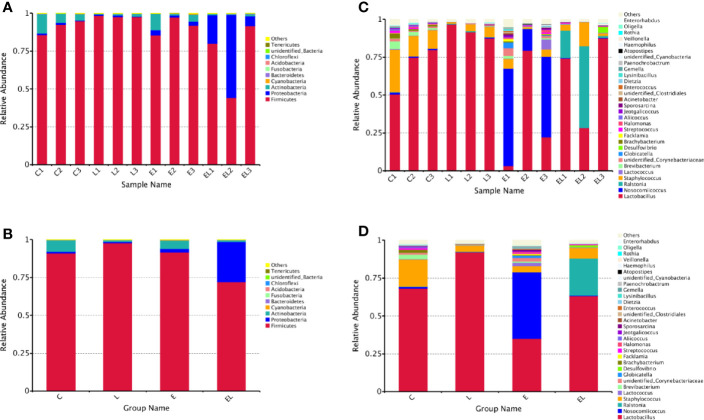
Relative abundance of the most preponderant (top 10 and 30) gut microbial taxa at phylum (top 10) and genus (top 30) levels for bacteria among four groups. **(A, C)** Relative abundance of gut microbiota in each sample at the phylum and genus levels. **(B, D)** Relative abundance of gut microbiota on the basis of the average number of each subfamily at the phylum and genus levels.

**Figure 5:**
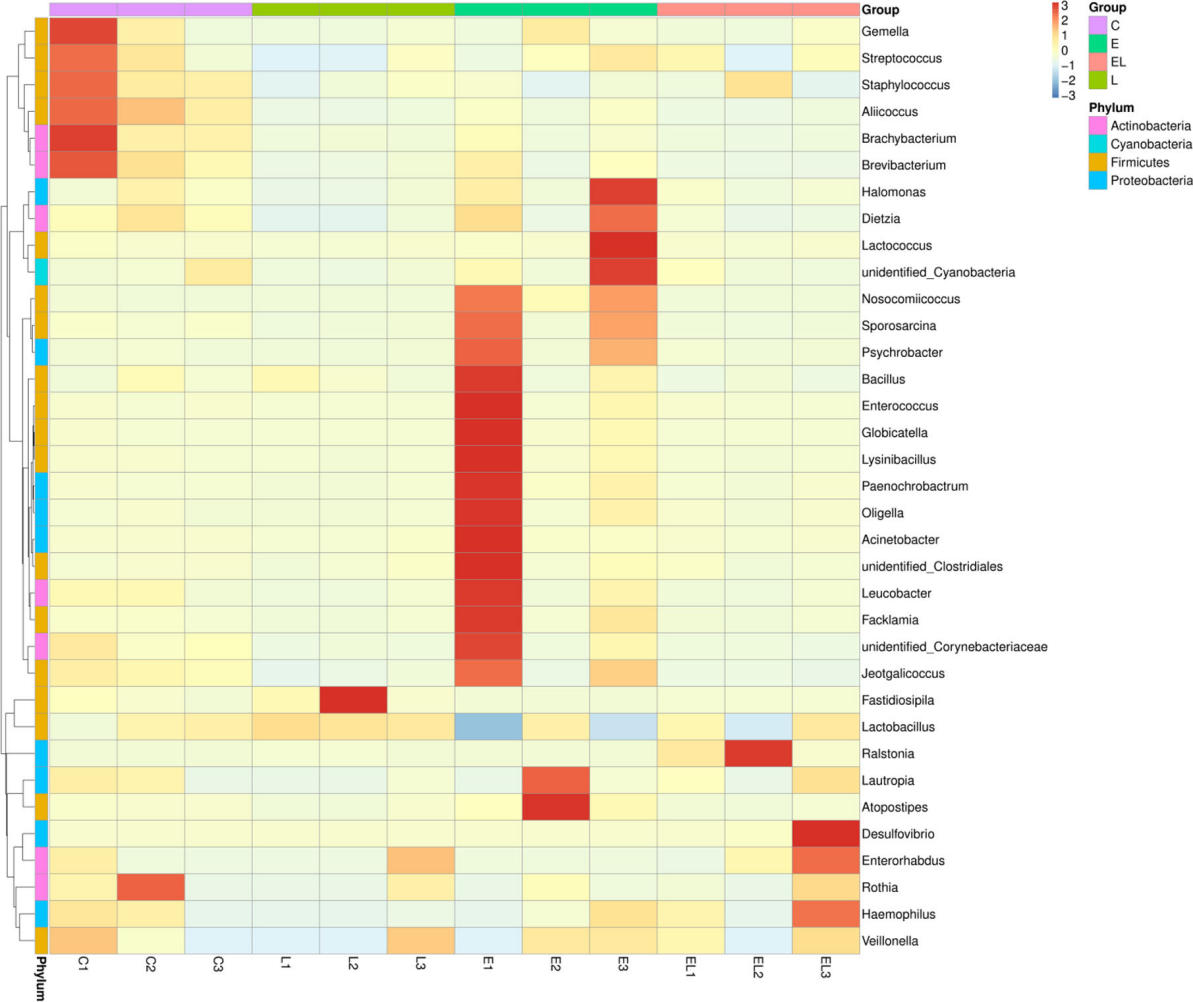
Heatmap of the relative abundance of bacterial communities at the genus level in each sample. Each color-block in the heatmap indicates the relative abundance of a bacterial genus in a sample.

The proportion of dominant phyla and genera were assessed by microbial taxa assignment in C, L, E, and EL groups (**Figure 4**). According to the phylum assignment result, phyla *Firmicutes* (91.08%, 97.82%, and 91.58%) and *Actinobacteria* (7.64%, 1.04%, and 5.51%) were the most preponderant bacteria in the mice of C, L, and E group, which accounted for approximately 97% of the taxonomic groups identified (**Figures 4A, B** **)**. Remarkably, the predominant phylum in the EL-group was *Firmicutes* (72.01%), whereas phylum *Proteobacteria* was subsidiary (26.69%), slightly different from the other groups. Other phyla such as *Fusobacteria*, *Acidobacteria*, *Chloroflexi*, *unidentified_Bacteria*, and *Tenericutes* were represented with a lower abundance. At the genus level, *Lactobacillus* (68.30% and 91.98%) was the most predominant bacterium in the mice of C-group and L-group followed by the *Staphylococcus* (17.92% and 4.07%), which together made up 85% and 95% of the overall bacterial composition, respectively (**Figures 4C, D** **)**. Moreover, *Nosocomiicoccus* (43.94%) and *Lactobacillus* (34.97%) were the most prevalent bacteria in the E-group, whereas *Lactobacillus* (63.23%) and *Ralstonia* (24.47%) were observed to be predominant in the EL-group. The relative richness of these bacteria was also displayed by a heat map produced by clustering analysis (**Figure 5**).

**Figure 6:**
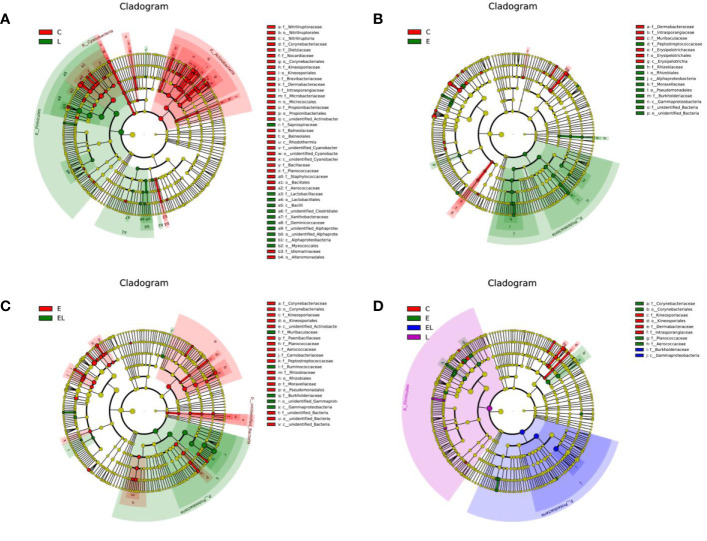
Differentially abundant phylotypes in different groups on the basis of LEfSe analysis. Cladogram obtained from LEfSe analysis displayed the different taxa in microbiota of different groups of mice. **(A)** Cladogram indicating the phylogenetic distribution of microbiota correlated with the C and L groups. **(B)** Cladogram indicating the phylogenetic distribution of microbiota correlated with the C and E groups. **(C)** Cladogram indicating the phylogenetic distribution of microbiota correlated with the E and EL groups. **(D)** Cladogram indicating the phylogenetic distribution of microbiota correlated with the C, E, EL and L groups. The colored circles from the inside to the outside represent different taxonomic levels (phylum, class, order, family, and genus levels). The yellow circles in the cladogram indicate the taxa with no evident differences.

**Figure 7:**
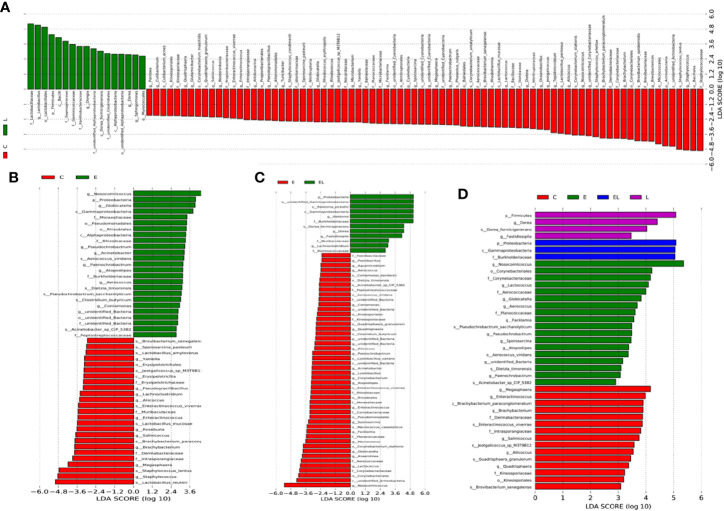
The differences in abundance between the different groups were evaluated using LDA scores. **(A)** The differences in abundance between the C and L groups. **(B)** The differences in abundance between the C and E groups. **(C)** The differences in abundance between the E and EL groups. **(D)** The differences in abundance between the C, E, EL and L groups. LDA scores > 2 was considered statistically significant.

To further compare the differences in intestinal microflora among the four groups, Linear discriminant analysis effect size (LEfSe) analysis coupled with Linear discriminant analysis (LDA) was performed for different classification levels (**Figures 6** and **7**). At the phylum level, *Firmicutes* was obviously more abundant in L-group than in the C-group, whereas the abundance of *Cyanobacteria* and *Actinobacteria* was lower. Additionally, the abundance of the *Proteobacteria* was significantly increased in E-group in comparison with C-group. At the genus level, *Lactobacillus* and *Sphingomonas* levels tended to be higher in the L-group than C-group, whereas the *Pantoea*, *Cutibacterium*, *Glutamicibacter*, *Turicibacter*, *Globicatella*, *Microbacterium*, *Facklamia*, *unidentified_Corynebacteriaceae*, *Brachybacterium*, and *Staphylococcus* showed the opposite trend (**Figures 6A** and **7A**). Moreover, a comparison of the E and C groups displayed a significant increase in the abundance of *Globicatella*, *Acinetobacter*, *Aerococcus*, and *Comamonas* as well as a distinct decrease in the abundance of *Roseburia* and *Megasphaera* (**Figures 6B** and **7B**). Meanwhile, the E-group was significantly enriched for *Paenibacillus*, *Aerococcus*, *Comamonas*, *Acinetobacter*, *Corynebacterium*, *Facklamia*, and *Globicatella* in comparison with EL-group (**Figures 6C** and **7C**).

## Discussion

In livestock industry, diarrhea is widely prevalent in juvenile animals, which is deemed as a crucial factor resulting in the reduction of global animal productivity (Pepin et al., 2004; Diao et al., 2020). Multiple measures have been performed to prevent diarrhea, but it still occurs from time to time. Recently, role of gut microbiota is revealed in the development of diarrhea (Huang et al., 2020). Therefore, the improvement of the intestinal microbial community structure may contribute to alleviate diarrhea (Yue et al., 2019). The significance of the *Lactobacillus* has been widely acknowledged as a result of its role in gut microbiota, metabolism, immunity, and health maintenance, but few reports have been published on the *Lactobacillus* from yaks inhabiting the Tibet Plateau (Li et al., 2018b; Wang et al., 2020). In this study, we analyzed the influence of *Lactobacillus* isolated from yaks on the gut microbiota and investigate whether it could improve the microbial community structure of mice with *E. coli*-induced diarrhea. Our results indicated that the *Lactobacillus* administration alleviated the intestinal microbial community of diarrheal mice colonized by *E. coli*.

Previous studies revealed that mammalian gut microbiota was dynamically varied during development and reached stability at maturity (Poroyko et al., 2011; David et al., 2014, Yang et al., 2020). Diarrheal diseases were widespread in the childhood of animals, which may be closely related to their immature gut microbiota (Wang et al., 2018b). Therefore, probiotics supplementation in the juvenile period of animals may reduce diarrhea by improving the structure in the intestinal microbial community (Wang et al., 2018a). Our study found the phylum *Firmicutes* as the most dominant bacteria in all samples regardless of the treatment. Consistent with previous observations, this phylum was also found to be widely distributed in camels, sheep, goats, and roe deer, indicating its important role in intestinal ecology and function (Li Z. et al., 2014; Zeng et al., 2017; Lei et al., 2018). The *Firmicutes* is responsible for digestion of cellulose and its richness in the gut contributes to meet the energy and nutrition requirements of animals in the growth and development process (Sun et al., 2016). Additionally, *Firmicutes* is mainly composed of gram-positive bacteria including *Lactococcus*, *Bacillus*, and *Lactobacillus* and most of them are perceived as beneficial bacteria, which are conducive to inhibit the proliferation of pathogenic bacteria and improve the intestinal environment (Garneau et al., 2008).

Importantly, our study also found a higher variation in some bacterial phyla and genera of different treatment groups, and this variation may play a crucial role in the intestinal ecosystem and function. *Cyanobacteria* comprises a great quantity of cyanotoxin-producing bacteria, posing a great threat to animal and human health (Carmichael, 1992). Wang et al. (2018b) observed that the proportion of *Actinobacteria* in the gut of diarrheal goat was significantly increased. Moreover, the synergy of *Actinobacteria* with one partner or host can easily be transformed into a pathogenic interaction with another (Miao and Davies, 2010). Mice in the L-group displayed increased *Firmicutes* and decreased *Cyanobacteria* and *Actinobacteria* abundance when compared to C-group, indicating a possible reduction in disease risk through *Lactobacillus* supplementation. Moreover, *Lactobacillus* and *Sphingomonas* were enriched in mice treated with *Lactobacillus*, whereas *Pantoea*, *Cutibacterium*, *Glutamicibacter*, *Turicibacter*, *Globicatella*, *Microbacterium*, *Facklamia*, *unidentified_Corynebacteriaceae*, *Brachybacterium*, and *Staphylococcus* were reduced in the control group. Previously, *Lactobacillus* had improved the intestinal mucosal immunity and interact with intestinal epithelial cells against entero-invasive *E. coli* (Johnson-Henry et al., 2007; Wang et al., 2019; Dong et al., 2019). Studies have also reported that supplementing diet with *Lactobacillus* daily can prevent non-alcoholic fatty liver disease by ameliorating the intestinal environment and attenuating inflammation in obese mice (Zhang et al., 2020a; Zhang et al., 2020b). Aside from improving immunity and regulating gut microbiota, *Lactobacillus* supplementation enhanced digestive enzyme activity and intestinal antioxidant ability benefiting the host (Chen F. et al., 2020; Zhang et al., 2020). *Sphingomonas* can degrade multiple organic matter, displaying the great application potential in environmental protection and industrial production (Leys et al., 2004). *Pantoea*, a gram-negative pathogenic bacterium, is associated with disease in plants, humans, and rarely in domestic animals (Silva-Rojas et al., 2012). Henker et al. (2020) indicated that *Pantoea* could induce fibrinonecrotic placentitis and abortions in mare. Zaccone et al. (2020) reported that *Pantoea* was closely related to bacteremia in humans. *Cutibacterium* was considered as skin flora contaminant, which can result in pericarditis with serious complications (Fakhri et al., 2020). Moreover, *Cutibacterium* was also closely related to multiple postoperative complications, including persistent postoperative pain, chronic inflammation, and endoprosthesis involving bacterial biofilms because it ubiquitously colonizes the skin and resides in various other locations in the human body (Patel et al., 2009; Achermann et al., 2013; Hudek et al., 2021). *Turicibacter* is a pro-inflammatory bacterium whose level rises during enteritis (Bretin et al., 2018). *Globicatella* was previously reported to associate with meningitis and bacteremia (Lau et al., 2006; Seegmuller et al., 2007). Furthermore, *Globicatella* was also observed in the purulent joint and lung infections in calves and sheep (Vandamme et al., 2001). *Microbacterium*, a novel bacterial pathogen, was also closely related to bacteremia (Hodgkin et al., 2000; Lau et al., 2002). *Facklamia* may be relevant to invasive disease such as meningitis and septicemia (Hughes, 2014; Parvataneni et al., 2015). *Corynebacteriaceae* can lead to endocarditis (Prada et al., 1994). *Brachybacterium* can cause bloodstream infection (Tamai et al., 2018). Most pathogenic *Staphylococcus* can produce coagulase, staphylolysin, enterotoxin, and toxic shock syndrome toxin1, resulting in fever, emesis, diarrhea, acute gastroenteritis, and even shock (Gemeinder et al., 2020; White et al., 2020). Moreover, *Staphylococcus* can invade the host through multiple ways and cause local and systemic infections as well as various invasive diseases such as pneumonia, meningitis, blood poisoning, and septic pyemia (Chen H. A. et al., 2020; Ranzani et al., 2020). Remarkably, *E. coli* infection significantly increased *Acinetobacter*, *Aerococcus*, and *Comamonas* levels and decreased *Roseburia* and *Megasphaera* content as compared to control group. *Acinetobacter*, a common opportunistic pathogen, is widely colonized in the digestive tract, skin, respiratory tract, and genitourinary tract, which can cause bacteremia, pneumonia, endocarditis, as well as urinary and skin infections (Go et al., 1994; Livermore and Woodford, 2006; Lima et al., 2020). *Aerococcus* can result in endocarditis and urinary tract infections (Yabes et al., 2018; Sous et al., 2019). *Comamonas* may be closely related to bacteremia (Liu et al., 2020a; Palacio et al., 2020). *Roseburia*, a butyrate-producing bacterium, can utilize sucrose, cellobiose, galactose, and glycogen (Duncan et al., 2002). *Megasphaera* was previously reported to produce short-chain fatty acids (SCFAs), displaying a positive regulatory effect to physiological functioning of gut and intestinal permeability (Kim et al., 2002). Conversely, *Lactobacillus* supplementation significantly reduced *Paenibacillus*, *Aerococcus*, *Comamonas*, and *Corynebacterium* levels in the ileum of mice induced by *E. coli*. Although reports of *Paenibacillus* infections are exceedingly rare, the infection may result in meningitis in some cases (Hunt et al., 2020). *Corynebacterium*, an acclimatized pathogen, can cause respiratory disease (Samies et al., 1986). This study conveyed a message that *Lactobacillus* supplementation resulted in an increase in beneficial bacteria and decreased pathogenic bacteria, whereas *E. coli* infection increased the ratio of harmful and beneficial bacteria. Additionally, *Lactobacillus* administration effectively ameliorate the microbial community structure of mice induced by *E. coli* and decreased the proportion of pathogenic bacteria.

## Conclusion

In summary, this study revealed that the gut microbiota in diarrheal mice induced by *E. coli* undergoes striking changes, characterized by an increased proportion of harmful bacteria. Conversely, *Lactobacillus* administration not only improves the microbial community structure of normal mice but also alleviates *E. coli*-induced diarrhea by mediating gut microbiota. These results expand our understanding of the potential benefits of *Lactobacillus* from yaks and convey an important message that *Lactobacillus* may be one of effective methods to attenuate diarrhea in yaks. Importantly, these findings also enriched the knowledge of disease prevention and control system in yaks. However, several limitations in this study need to be noticed, such as individual variation, experimental environment, and small sample size.

## Data Availability Statement

The datasets presented in this study can be found in online repositories. The names of the repository/repositories and accession number(s) can be found below: https://www.ncbi.nlm.nih.gov/, PRJNA665922.

## Ethics Statement

The animal study was reviewed and approved by the ethics committee of Tibet Agriculture & Animal Husbandry University.

## Author Contributions 

QW, HZ, HD and AL conceived and designed the experiments. AL, YC, QW, HZ, HD and BL contributed reagents, materials, and analysis tools. HD wrote the manuscript. HZ, QW, MI, KM and TJ revised the manuscript. All authors contributed to the article and approved the submitted version.

## Funding

This research was funded by the Key research, development, and transformation program of Tibet autonomous region (XZ201902NB05), the Key laboratory of clinical veterinary medicine in Tibet, and the Research and demonstration of technologies for prevention and control of major infectious diseases in characteristic livestock (XZ201901NA02).

## Conflict of Interest

The authors declare that the research was conducted in the absence of any commercial or financial relationships that could be construed as a potential conflict of interest.
